# COVID-19 and Suicide Mortality in a Population-Based Medico-Legal Registry: An Interrupted Time-Series and Changepoint Analysis from Southeast Romania

**DOI:** 10.3390/diagnostics16172736

**Published:** 2026-08-26

**Authors:** Alin Ionut Piraianu, Anisia-Luiza Culea-Florescu, Elena Stamate, Ana Fulga, Doriana Iancu, Octavian Stefan Patrascanu, Iuliu Fulga

**Affiliations:** 1 Research Centre in the Medical-Pharmaceutical Field, Faculty of Medicine and Pharmacy, “Dunarea de Jos” University of Galati, 800216 Galati, Romania; alin.piraianu@ugal.ro (A.I.P.); ana.fulga@ugal.ro (A.F.); c_doriana@yahoo.com (D.I.); octav974@gmail.com (O.S.P.); iuliu.fulga@ugal.ro (I.F.); 2 Research Center for Electronics, Information Technology and Communications, Faculty of Automation, Computers, Electrical and Electronic Engineering, “Dunarea de Jos” University of Galati, 800201 Galati, Romania

**Keywords:** suicide, COVID-19, SARS-CoV-2, interrupted time series, segmented regression, changepoint detection, forensic medicine, medico-legal registry, Romania

## Abstract

**Background:** Interrupted time-series studies have not shown a consistent increase in suicide mortality during COVID-19, but civil mortality registries cannot ascertain SARS-CoV-2 infection individually. We evaluated pandemic-related change in suicide mortality and the feasibility of integrating infection ascertainment into medico-legal surveillance. **Methods:** We analysed 395 suicide deaths in Galați and Brăila counties, Romania (January 2018–December 2024), where mandatory forensic autopsy ensures exhaustive ascertainment. The pre-specified primary analysis was seasonally adjusted segmented Poisson regression in January 2018–December 2022 with an onset in March 2020; the period 2023–2024 was excluded as incompletely ascertained. Changepoint detection and unadjusted period comparisons were descriptive. Infection was classified as laboratory-confirmed (documented ante-mortem RT-PCR) or possible/probable (compatible autopsy findings); no post-mortem testing was performed. **Results:** The level change at onset was not significant (IRR 1.29, 95% CI 0.80–2.08; *p* = 0.302), nor was the slope change (IRR 1.01, 95% CI 0.98–1.04; *p* = 0.639); power was 80% only against IRR ≥ 1.98. Posterior probabilities were 0.85 for any increase and 0.035 for a doubling. The unadjusted comparison (6.00 vs. 4.62 deaths/month; IRR 1.30, 95% CI 1.04–1.63) was descriptive; no changepoint coincided with onset. Among 140 deaths in 2020–2021, 34 (24.3%) met ascertainment criteria, 21 (15.0%) laboratory-confirmed. **Conclusions:** The data exclude a doubling of suicide mortality but not a modest increase: a regional surveillance signal, not a causal finding. Infection ascertainment proved feasible within routine medico-legal investigation, yielding a reproducible framework for other registries, an Eastern European series for future meta-analyses, and a proof-of-concept for a standing forensic surveillance capacity adaptable to future epidemics.

## 1. Introduction

From the earliest weeks of the COVID-19 pandemic, mental-health researchers warned that the combination of social isolation, economic disruption, bereavement, and reduced access to healthcare could increase suicide risk [1,2,3]. The empirical evidence has proved both more reassuring and more complex. Large multi-country interrupted time-series (ITS) studies demonstrated that suicide rates did not increase during the first months of the pandemic compared with modelled expectations and even declined in several settings; subsequent analyses covering the first 9–15 months largely confirmed stable or decreasing trends, with only isolated exceptions [4,5,6,7,8].

Published studies rely predominantly on civil death-registration systems, which record the fact and manner of death but do not establish whether the deceased was infected with SARS-CoV-2 at the time of death. Consequently, the relationship between SARS-CoV-2 infection and suicide—whether mediated through neuropsychiatric sequelae, the psychological impact of diagnosis and isolation, or pre-existing psychiatric vulnerability—has remained largely inferential at the population level. Small forensic case series have documented suicide among SARS-CoV-2-positive individuals, but the available literature remains scarce [9,10].

Previous population-based registry studies have quantified pandemic-associated changes in suicide mortality, whereas forensic studies have examined SARS-CoV-2 infection among suicide decedents. Evidence integrating population-level temporal analysis with individual-level infection ascertainment based on medico-legal documentation and autopsy findings within the same complete case series remains limited.

Population-based forensic registries offer a unique opportunity to address this knowledge gap [9,10,11,12]. Because forensic autopsy is mandatory for all violent and suspected violent deaths under Romanian criminal procedure [13], these registries provide exhaustive population coverage within a defined geographical area. Moreover, unlike civil mortality databases, they allow SARS-CoV-2 status to be assessed through ante-mortem clinical and laboratory documentation incorporated into medico-legal records and through post-mortem morphological findings at forensic autopsy [9,10]. Consequently, forensic registries permit both interrupted time-series analyses of suicide mortality and direct estimation of ascertained SARS-CoV-2 infection among suicide decedents, providing evidence that cannot be obtained from conventional mortality statistics alone [11,12,14,15,16,17,18].

Segmented regression and changepoint detection are complementary rather than interchangeable. Segmented Poisson regression tests a hypothesis specified a priori—that the rate changed at a stated date—and quantifies the change; changepoint algorithms make no such assumption and search the series for structural breaks wherever they occur. Applying both allows an a priori-dated effect to be examined against a data-driven alternative [19,20,21,22,23,24,25,26].

The novelty of the present work lies in the data source: we analysed a complete regional medico-legal suicide registry from southeastern Romania spanning the pre-pandemic, pandemic, and early post-pandemic periods, in which every suicide within the catchment area is captured by mandatory autopsy and infection status is documented in the same record. Our objectives were threefold: (i) to quantify the immediate and sustained change in monthly suicide counts following the onset of the COVID-19 pandemic using seasonally adjusted segmented Poisson regression [19,20,21,22]; (ii) to identify structural breaks independently using changepoint detection [23,24,25,26]; and (iii) to determine the prevalence and characteristics of forensically ascertained SARS-CoV-2 infection among suicide decedents. We hypothesised that integrating interrupted time-series methodology with comprehensive forensic registry data would provide a more complete assessment of the pandemic’s association with suicide mortality than analyses relying exclusively on civil mortality records. Beyond these objectives, the study was designed to establish whether infection ascertainment can be embedded in routine medico-legal investigation in a reproducible form, a question of operational relevance for forensic surveillance during future public-health emergencies (Section 4.7).

**Figure 1:**
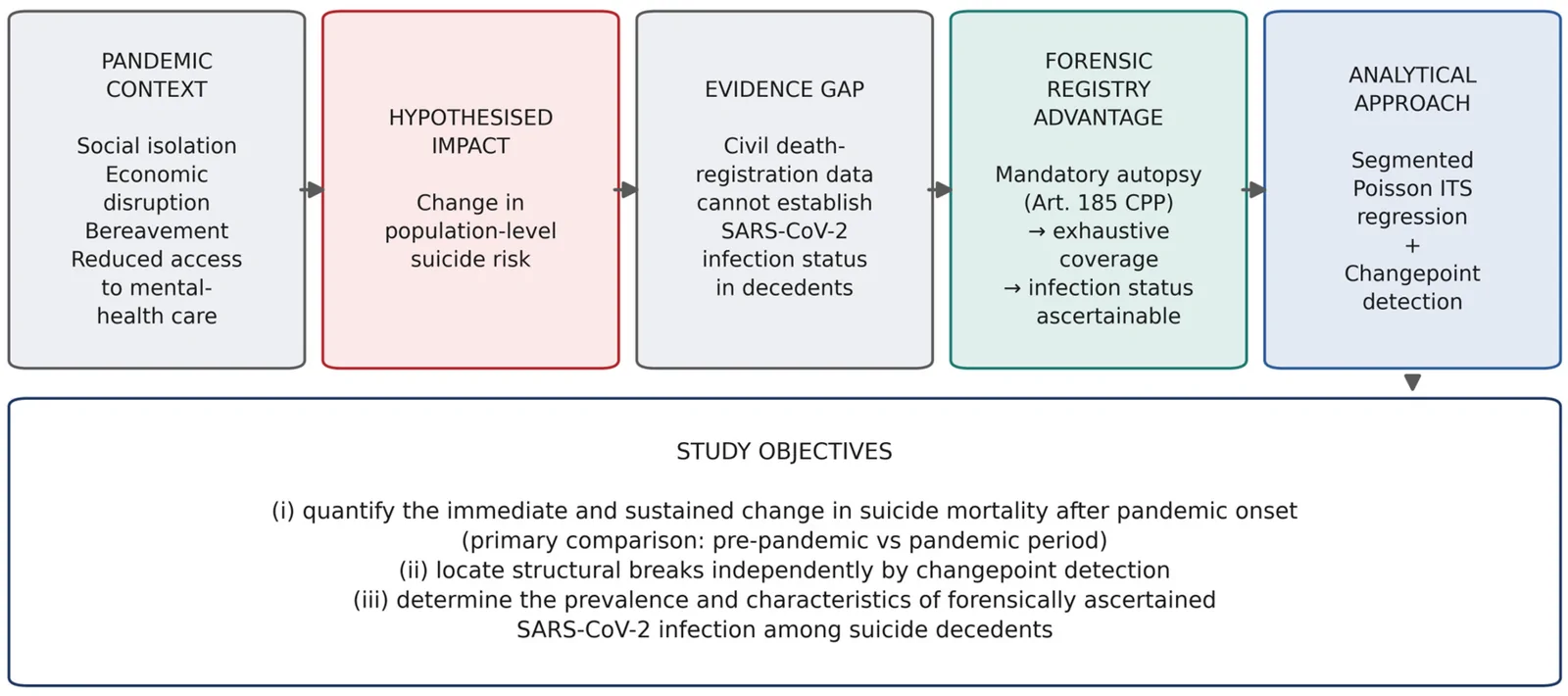
Conceptual overview of the study rationale, the evidence gap addressed, and the analytical framework.

A conceptual overview of the study rationale and analytical framework is presented in Figure 1.

## 2. Materials and Methods

### 2.1. Design, Setting and Data Source

This was a retrospective, population-based ITS study using the complete medico-legal suicide registry of Galați and Brăila counties, southeastern Romania. At the 2021 Population and Housing Census, the two counties had a combined resident population of 778,344 (Galați 496,892; Brăila 281,452), of whom 55.4% lived in urban settlements [27]. All 395 consecutive suicide deaths between 1 January 2018 and 31 December 2024 were included—84 consecutive calendar months. Records were fully anonymised. The exhaustive forensic ascertainment described in Section 2.2 yields a population-based count series rather than a sample.

### 2.2. Medico-Legal Framework in Romania

Under Article 185 of the Romanian Code of Criminal Procedure, a medico-legal autopsy is ordered by the criminal investigation body or by the court in every case of violent death, of death suspected of being violent, of death of unknown cause, and wherever there is reasonable suspicion that the death was caused directly or indirectly by an offence. In practice, the ordering authority is the prosecutor, who issues a written ordinance; suicide falls within the category of violent death and is therefore autopsied without exception.

The examination is performed exclusively by a forensic pathologist (forensic pathologist) employed within the network of medico-legal institutions organised under Government Ordinance no. 1/2000, as republished, and not by a hospital anatomical pathologist; Article 185(4) of the Code requires the autopsy to be carried out within a medico-legal institution. Procedural conduct, the content of the report and the required investigations are governed by the Procedural Norms approved by Joint Order of the Minister of Justice and the Minister of Health no. 1134/C/255/2000. Additional specialists from other medical fields may be co-opted at the forensic pathologist’s request, with the express exclusion of the physician who treated the deceased (Article 185(5)). The forensic pathologist issues an expert report stating identity, the manner of death, the medical cause of death, and the traumatic lesions and their causal relationship to death (Article 185(8)). Manner of death—and hence the classification of a death as suicide—is therefore a medico-legal determination made on autopsy, supported by scene investigation and toxicology, rather than a category assigned administratively at death registration.

During the pandemic, the handling of deceased persons with suspected or confirmed SARS-CoV-2 infection followed a national protocol issued by the Ministry of Health (Order no. 436/2020, replaced by Order no. 570/2020 and subsequently amended by Order no. 487/2021) [28]. The protocol required that all suspected cases be treated as confirmed for biosafety purposes, restricted medico-legal autopsy of such cases to those ordered by the criminal investigation authorities, and prescribed the sampling, ventilation, personal-protection and body-handling measures to be applied. Suicides, being violent deaths, continued to be autopsied throughout the pandemic; consequently, ascertainment of the suicide series itself was unaffected by changes in autopsy practice (Section 2.3).

### 2.3. Outcome, Exposures and SARS-CoV-2 Case Definition

The outcome was the monthly count of suicide deaths. The principal exposure was the pandemic onset, fixed in March 2020, the month Romania declared a national state of emergency (16 March 2020); this defines month 27 of the 84-month series.

SARS-CoV-2 infection status was classified in two tiers. A decedent was classified as laboratory-confirmed when a positive ante-mortem SARS-CoV-2 RT-PCR result was documented in the clinical records incorporated into the medico-legal file. No post-mortem laboratory testing for SARS-CoV-2 was performed in this series. Decedents without documented ante-mortem laboratory confirmation but showing pulmonary findings morphologically compatible with COVID-19 at forensic autopsy were classified as possible/probable, because these findings are suggestive but not specific for SARS-CoV-2 infection.

### 2.4. Interrupted Time-Series Model

The primary analysis was restricted to the pre-pandemic and pandemic periods (January 2018–December 2022, 60 months). This restriction was adopted because case ascertainment for 2023–2024 is demonstrably incomplete (Section 4.6), so retaining those months as a main comparator would allow an administrative artefact to drive the estimates. Data for 2023–2024 are presented as a supplementary period only, with the caveat stated at every point of use, and are reported in the Supplementary Materials.

The seasonally adjusted segmented Poisson regression specified below is the pre-specified primary analysis; the unadjusted period rate-ratio comparison (Section 2.6) and the changepoint analyses (Section 2.5) are secondary and descriptive, and no substantive conclusion of the paper rests on either. The restriction of the series to January 2018–December 2022 is based on demonstrably incomplete registration of 2023–2024 forensic files at the date of data extraction (Section 4.6), a property of the data source and independent of the estimates the restriction produces. The pre-specified model is reported below as the primary analysis with its non-significant estimate, and the unadjusted comparison is reported as descriptive throughout.

Monthly counts *Y*t were modelled by segmented Poisson regression as *Y*t ~ Poisson(μt) with log(μt) = β0 + β1·*t* + β2·*D*t + β3·(*t* − *t*0)·*D*t + β4·sin(2π*m*/12) + β5·cos(2π*m*/12), where *t* = 1, …, 60 indexes calendar month from January 2018, *D*t = 0 before March 2020 and 1 from March 2020 onward, *t*0 = 27 (March 2020) and *m* is the calendar month number. β2 is the immediate level change at onset and β3 the change in slope after onset; β4 and β5 parameterise a single annual harmonic. Coefficients were exponentiated to incidence rate ratios (IRRs) with Wald 95% confidence intervals. We assessed overdispersion using the Pearson dispersion statistic; we fitted a negative-binomial model as a sensitivity check. The segmented-regression specification followed established ITS guidance [19,20,21,22].

A model with the natural logarithm of the monthly resident population as an offset was pre-specified so that estimates could be expressed as rates rather than counts, and that model was fitted. The denominator series was constructed by log-linear interpolation between the resident-population totals recorded at the two national censuses bracketing the study window—857,379 for Galați and Brăila counties combined at the October 2011 census and 778,344 at the December 2021 census [27]—and is reported in full in Supplementary Table S3. The census totals were used directly rather than table POP107D. POP107D records population by domicile, an administrative registration figure that runs higher than resident population because people commonly retain their domicile registration after moving away, and it is not the appropriate series for a resident-population offset. With this offset, the level change at pandemic onset was IRR = 1.29 (95% CI 0.80–2.08, *p* = 0.302), the same estimate as the count-based primary model; the full coefficient table is given in Supplementary Table S2. This is the expected result rather than a coincidence: the resident population of the two counties declines close to log-linearly over the five-year analysis window, and a log-linear denominator entered as an offset is absorbed by the intercept and the linear trend term, leaving the level and slope coefficients unchanged.

Three sensitivity analyses were pre-specified: a negative-binomial specification, refitting the primary model with the series truncated at December 2023 (because counts for the most recent complete year are most exposed to registration delay), and the population-offset model described above. On the series truncated at December 2023 (*n* = 72 months), the level change at onset was IRR = 1.54 (95% CI 0.98–2.43, *p* = 0.061). The negative-binomial specification on the restricted series returned coefficients numerically identical to the Poisson fit, with the dispersion parameter estimated near zero—the expected degenerate result when the data show no overdispersion beyond what the Poisson model already accommodates, consistent with the Pearson dispersion statistic of 0.80 reported for the primary model. Because the validity of the Wald interval for the level change depends on the absence of residual serial correlation, we also examined autocorrelation in the Pearson residuals of the primary model using the Durbin–Watson statistic and the Ljung–Box test at lags 1–12. The Durbin–Watson statistic was 2.20, close to the value of 2 expected under no autocorrelation, and the Ljung–Box test found no significant autocorrelation at any lag from 1 to 12 (all *p* ≥ 0.08). The Wald interval reported for the level change is therefore not compromised by residual serial correlation.

**Figure 2:**
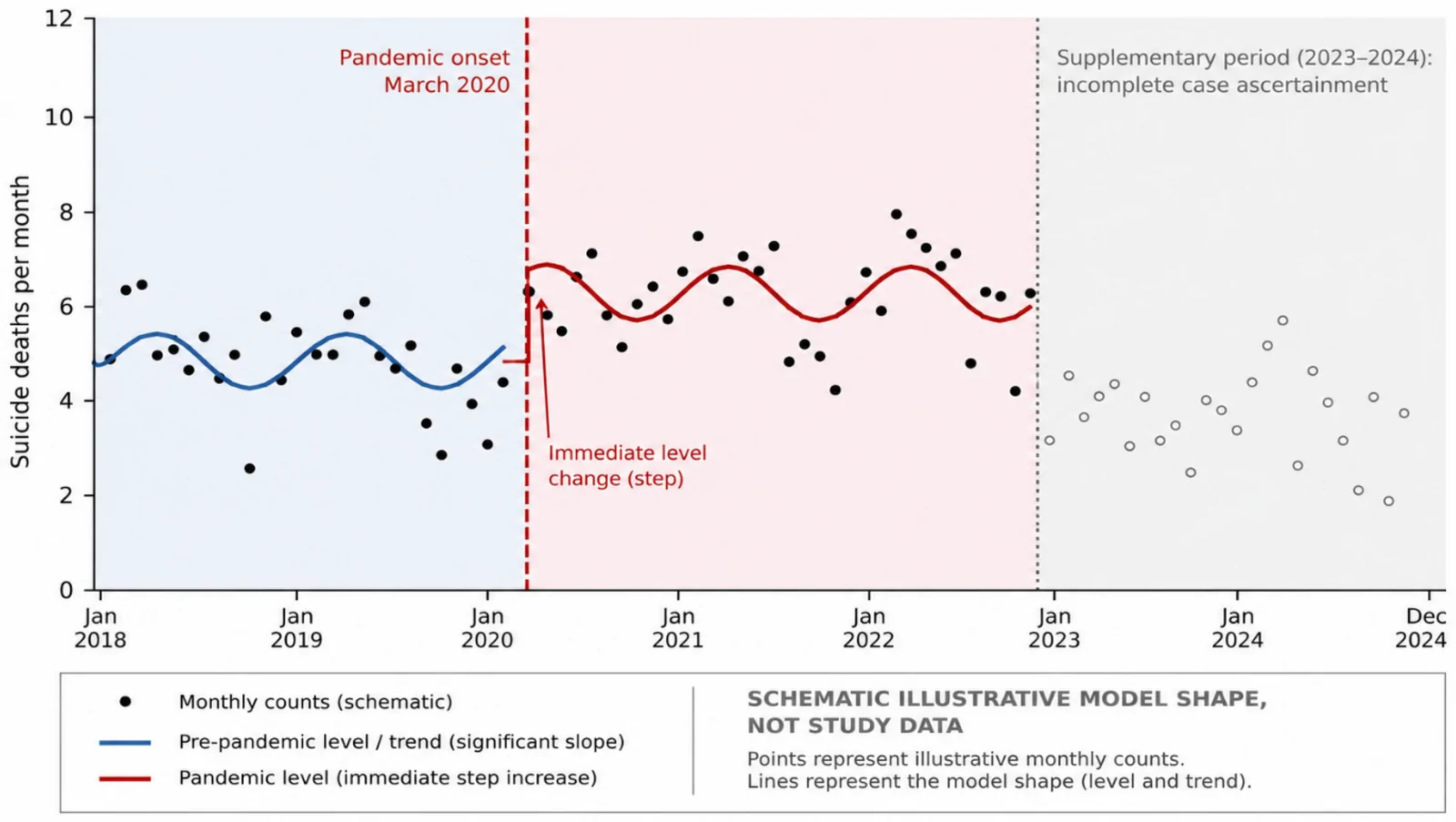
Schematic representation of the segmented interrupted time-series model, illustrating the pre-pandemic level, the step changes at the March 2020 onset, and the subsequent slope. The figure does not display observed data; the hatched area indicates the supplementary period excluded from the primary analysis.

The conceptual structure of the interrupted time-series model is shown in Figure 2.

### 2.5. Changepoint Detection/Methods

As an assumption-light, data-driven cross-check on the location of structural change, changepoint analysis was performed on the monthly series using the Pruned Exact Linear Time (PELT) algorithm and binary segmentation [23,24,25]. PELT identifies the number and position of changepoints by penalised cost minimisation in linear time; binary segmentation provides a complementary fixed-number-of-breaks solution. Detected breakpoints were compared with the a priori pandemic onset. Break dates are reported throughout as the first month of the new segment.

Both algorithms were applied to the mean-shift cost function of the rupture’s library. For PELT, the penalty term determines the number of breaks returned and must therefore be reported. The analysis used the following BIC-type penalty:

pen = 3 \cdot Var(x) \cdot log(n) = 80.6

where *n* = 84 months and the sample variance was 6.07.

A penalty sweep from 0.5× to 8× this value showed that the January 2023 break was retained at 0.5× and 1×, whereas no break was retained at 2× or above.

Binary segmentation was run with the number of breakpoints fixed at two, chosen a priori to correspond to the two candidate transitions in the series. Because the primary analysis is confined to 60 months, both algorithms were additionally run on the restricted series (January 2018–December 2022), with the penalty recomputed by the same rule from the variance and length of that series; those results are reported in Section 3.4. A break at January 2023 lies immediately beyond the terminal boundary of the restricted series and is by construction not detectable there, so the restricted run bears only on the pandemic-onset question. Because the solution returned by PELT depends on the penalty, and because higher penalties retain only a late-series break, the changepoint results are reported as a consistency check on the segmented-regression model rather than as independent corroboration of the March 2020 onset.

### 2.6. Period Comparisons and Decedent Profiling

The period comparison contrasted the pre-pandemic period (January 2018–February 2020) with the pandemic period (March 2020–December 2022). It is a descriptive, unadjusted comparison of two mean monthly counts and is reported as such. It assumes a constant rate within each period and makes no allowance for the underlying time trend, for seasonality, or for serial correlation between adjacent months; its confidence interval is the standard two-rate Poisson interval, exp(log RR ± 1.96·√(1/*D*1 + 1/*D*2)) and therefore reflects sampling variation in the two death totals alone. It is not a more robust alternative to the segmented model and is no longer described as one. Comparisons involving the supplementary period (January 2023–December 2024) are reported separately and are not used to support any substantive conclusion. Decedents with ascertained SARS-CoV-2 infection were compared with the remainder on age, sex, residence, method and documented psychiatric history, with the laboratory-confirmed and possible/probable groups tabulated separately.

### 2.7. Software, Ethics and Reporting

Counts and period statistics were derived in Python 3.12.3 (pandas 2.2.3); segmented Poisson and negative-binomial models were fitted with statsmodels 0.14.4 (GLM, Poisson and NegativeBinomial families, log link) and changepoint analysis with the *ruptures* 1.1.9 library [26]; figures were produced with Matplotlib 3.10.8. The monthly count series from which every estimate reported in this paper is derived is provided as Supplementary Table S3, and the complete unedited output of every model fitted is provided as Supplementary Table S4, so that each figure in Tables 2–4 can be checked independently.

The study used anonymised medico-legal records under the legal mandate for forensic autopsy (Article 185 of the Code of Criminal Procedure), and the Declaration of Helsinki (2013); individual consent was not applicable. The study was approved by the Ethics Committee of the Brăila County Emergency Clinical Hospital, Brăila, Romania, approval no. 18 dated 30 July 2026. Reporting follows the STROBE statement [29].

**Figure 3:**
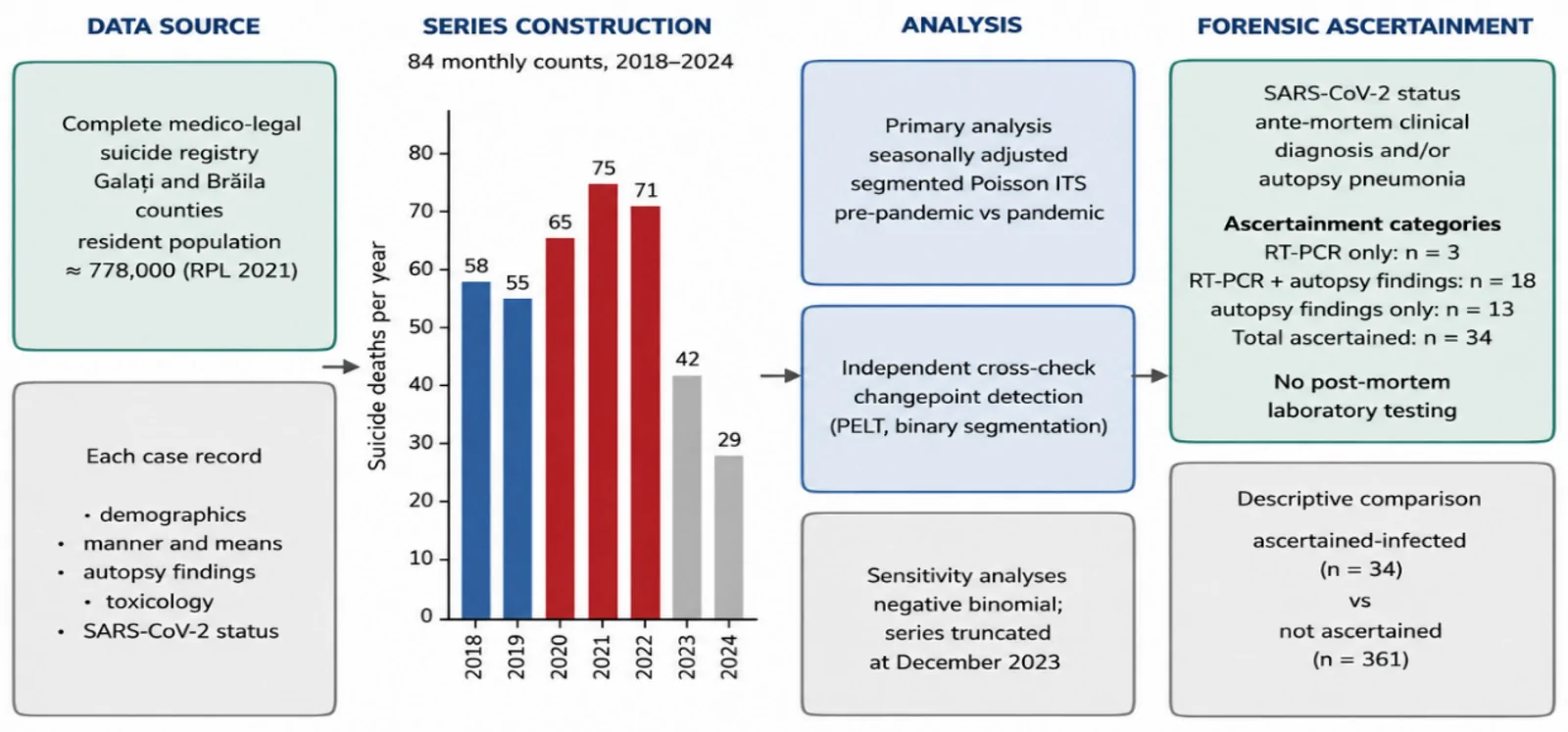
Study workflow showing the registry data source, construction of the 84-month count series, analytical strategy, and forensic ascertainment of SARS-CoV-2 status. Annual counts correspond to the observed data reported in Table 1; grey bars indicate the supplementary period excluded from the primary analysis. Blue bars indicate the pre-pandemic period and red bars the primary pandemic period.

The overall study workflow is illustrated in Figure 3.

## 3. Results

### 3.1. Overall Counts and Seasonality

Across 84 months, 395 suicide deaths were recorded (mean 4.70/month). Annual counts rose from 58 (2018) and 55 (2019) to a peak of 75 (2021), then declined to 42 (2023) and 29 (2024) (Table 1). A spring peak was evident, with the highest counts in April and May and the lowest in January, although only one of the two harmonic terms reached significance (Section 3.2), so the seasonal pattern is described here as consistent with, rather than as confirming, the long-recognised seasonality of suicide [17].

### 3.2. Segmented Poisson Regression

On the pre-specified primary model, fitted to the series restricted to January 2018–December 2022, the immediate change in the suicide rate at pandemic onset was not statistically significant: level IRR = 1.29 (95% CI 0.80–2.08, *p* = 0.302) (Table 2). There was no significant post-onset slope change (IRR = 1.01, 95% CI 0.98–1.04, *p* = 0.64) and no significant underlying pre-pandemic trend (*p* = 0.78). The Pearson dispersion statistic was 0.80, indicating no overdispersion; the negative-binomial sensitivity model returned coefficients numerically identical to the Poisson fit. Of the two seasonal harmonic terms, only the cosine term reached conventional significance (*p* = 0.046); the sine term did not (*p* = 0.352); the spring peak is therefore only partially supported by the model and is interpreted with corresponding caution throughout. The population-offset model specified in Section 2.4 returned a level change of IRR = 1.29 (95% CI 0.80–2.08, *p* = 0.302), numerically identical to the count-based primary model.

This restricted-series estimate differs from the 84-month, count-based model reported at first submission (level IRR = 1.67, 95% CI 1.09–2.56, *p* = 0.018). This difference results from removing the 2023–2024 months, in which ascertainment is demonstrably incomplete (Section 4.6); those months depress the post-onset average and thereby inflate the apparent step at onset when retained. The restricted estimate is the one the authors regard as valid, and the pre-specified model is reported as the primary analysis irrespective of its statistical significance.

The unadjusted period comparison of Section 3.3 yields a numerically almost identical point estimate (IRR = 1.30) with a much narrower confidence interval. The two approaches produced similar point estimates (IRR 1.29 vs. 1.30), but the segmented model yields substantially greater uncertainty because it must account for the underlying temporal trend, the post-onset slope change and seasonality, none of which the unadjusted comparison estimates. On the log scale, the standard error of the level change is 0.244 in the segmented model against 0.115 in the unadjusted comparison, a 4.5-fold difference in variance. The segmented model must estimate the step jointly with an underlying trend and a post-onset slope change with which it is strongly collinear over a 60-month window in which the break falls at month 27, and it must accommodate an annual harmonic; the unadjusted comparison assumes the rate is flat and aseasonal within each period and so attributes none of the between-month variability to those sources. The narrower interval reflects stronger assumptions, not greater robustness, and the wider interval reflects what the study design warrants.

The corresponding precision statement is that with 120 pre-pandemic and 204 pandemic-period deaths, the primary model had 80% power at α = 0.05 (two-sided) to detect a level change of IRR ≥ 1.98 or ≤0.50, and approximately 19% power against a true IRR of 1.30. An effect of the magnitude suggested by the raw period means was therefore never detectable in a series of this length. The non-significant result should accordingly be read as uninformative about level changes below roughly a doubling, and not as evidence that no change occurred.

The coefficients below are those of the pre-specified primary model fitted to the restricted 60-month series (January 2018–December 2022). The population-offset model of Section 2.4 and the sensitivity model truncated in December 2023 are reported in Supplementary Table S2; the full unedited software output for every model is given in Supplementary Table S4 and the monthly count series in Supplementary Table S3.

Because a dichotomised significance statement conveys little about a series of this length, the information in the primary estimate was additionally expressed as a posterior distribution for the level change, computed from the fitted level-change coefficient of the primary model (0.252, standard error 0.244; Table 2) under a flat prior on the log scale. The posterior probability that the level change exceeded unity is 0.85; that it exceeded 1.20, 0.61; that it exceeded 1.30—the magnitude implied by the raw period means—0.48; and that it reached a doubling, 0.035. The 90% credible interval is 0.86–1.92. On the sensitivity fit truncated in December 2023, the corresponding probabilities are 0.97, 0.86, 0.77 and 0.13, with a 90% credible interval of 1.05–2.26. The registry therefore leans towards a modest pandemic-period increase, is indeterminate as to whether that increase reached 30%, and is close to incompatible with a doubling of suicide mortality of the kind projected in the early pandemic literature. Full posterior summaries are given in Supplementary Table S6.

### 3.3. Period Rate Ratios

As a descriptive summary of the raw data, the pandemic period averaged 6.00 deaths per month against 4.62 pre-pandemic, an unadjusted rate ratio of 1.30 (95% CI 1.04–1.63; *p* = 0.023) (Table 3). This comparison is reported for completeness and is not used to support any inferential claim, for the reasons set out in Section 2.6. Read together with the primary model, the informative observation is that the two-point estimates agree (1.30 against 1.29) and that the primary model, which accounts for the structure of the series, cannot distinguish that estimate from no change. Supplementary Table S1 provides comparisons involving the supplementary period and detailed methodological information. In brief, the 2023–2024 mean of 2.96 deaths per month is roughly half the pandemic mean; the magnitude and abruptness of that decline exceed what is epidemiologically plausible over two years, and it is attributed to incomplete ascertainment of recently registered forensic cases rather than to a true fall in suicide mortality. No conclusion of this paper rests on it.

### 3.4. Changepoint Detection Results

Changepoint analysis did not provide independent evidence of a structural break at pandemic onset (Table 4). On the full 84-month series, binary segmentation was run with two breakpoints in February 2020—one month before the a priori onset—and January 2023, partitioning the series into a pre-pandemic level of ≈4.52 per month, a pandemic level of ≈6.03 per month, and a subsequent level of ≈2.96 per month. PELT, at the penalty reported in Section 2.5 (pen = 80.6), returned only a break in January 2023 and did not identify a break at pandemic onset; at higher penalties (2×–8× the BIC rule) no break at all was retained. On the restricted 60-month series, from which the January 2023 break is by construction excluded, PELT returned no break at the standard penalty (pen = 59.4, computed by the same rule from this series’ variance and length) and returned a break at February 2020 only when the penalty was halved; binary segmentation with one breakpoint returned a single break at February 2020, again one month before the a-priori onset, partitioning the series into a pre-pandemic level of ≈4.52 per month and a subsequent level of ≈6.03 per month. Because the two algorithms disagree on the full series, because the number of breaks returned by PELT is a function of the penalty chosen, and because the binary-segmentation solution is constrained to return the number of breaks it is given, the changepoint analysis is reported as a consistency check that neither confirms nor refutes the a priori onset.

### 3.5. Forensically Ascertained SARS-CoV-2 Among Decedents

Thirty-four of 395 decedents met either the laboratory-confirmed or the possible/probable SARS-CoV-2 ascertainment criterion. Because all 34 ascertained cases occurred in 2020 and 2021, we report two denominators, and the second supersedes the first as the epidemiologically meaningful figure: 8.6% of the full 84-month series (34/395) and 24.3% of the 140 suicide deaths recorded in 2020–2021 (34/140). The two ascertainment tiers are reported separately throughout and are not pooled in any inferential statement. Twenty-one decedents had a documented positive ante-mortem SARS-CoV-2 RT-PCR result and were classified as laboratory-confirmed: 5.3% of the whole series and 15.0% of deaths in 2020–2021. Of these, 18 also showed pulmonary findings morphologically compatible with COVID-19 at autopsy, whereas three had laboratory confirmation without such findings. A further 13 decedents had compatible pulmonary findings at autopsy without documented ante-mortem laboratory confirmation and were classified as possible/probable, corresponding to 9.3% of deaths in 2020–2021; because these findings are suggestive but not specific for SARS-CoV-2, this group carries a materially different evidential weight from the laboratory-confirmed group. Overall, compatible pulmonary findings were recorded at autopsy in 31 decedents. No decedent underwent post-mortem laboratory testing for SARS-CoV-2.

All 34 ascertained cases occurred in 2020 and 2021 (17 in each year), with no case meeting either criterion recorded thereafter. This distribution reflects both the epidemiology of the period and the cessation of routine ante-mortem testing, and the absence of cases after 2021 should not be read as an absence of infection among later decedents.

Table 5 compares the two ascertainment tiers with each other and with the decedents in whom SARS-CoV-2 was not ascertained. Taken together, the 34 ascertained decedents had a similar age and sex distribution to the remainder, a somewhat lower rural proportion (61.8% vs. 74.2%) and a higher prevalence of documented psychiatric history (58.8% vs. 29.4%), the latter corresponding to 20 of 34 ascertained decedents against 106 of 361 among the remainder. With 21 laboratory-confirmed and 13 possible/probable cases, all subgroup contrasts are descriptive and hypothesis-generating; no significance test is reported, and none of these contrasts should be interpreted as evidence of an association between SARS-CoV-2 infection and psychiatric morbidity. The psychiatric-history contrast does not depend on pooling the two tiers: it holds separately in each. Documented psychiatric history was present in 12 of the 21 laboratory-confirmed decedents (57.1%) and in 8 of the 13 possible/probable decedents (61.5%), both markedly higher than the 29.4% among the not-ascertained group, so the enrichment is not concentrated in the morphology-only tier where ascertainment bias would be strongest but is present at a similar magnitude in the tier based on laboratory confirmation.

## 4. Discussion

### 4.1. Principal Findings

This population-based forensic interrupted time-series study yielded three principal findings on the restricted pre-pandemic/pandemic series. First, the pre-specified, seasonally adjusted segmented regression did not demonstrate a statistically significant immediate increase in suicide deaths at pandemic onset (level IRR = 1.29, 95% CI 0.80–2.08, *p* = 0.302). The raw period means are numerically higher during the pandemic period, and the unadjusted rate ratio derived from them agrees with the model’s point estimate. Still, that comparison carries assumptions the design does not support and cannot substitute for the primary analysis. The honest reading of the first finding is that this registry is compatible with a modest pandemic-period increase and equally compatible with none: the confidence interval spans a 20% decrease to a doubling, and the study had only about 19% power against an effect of the size the raw means suggest. Expressed as a posterior distribution rather than as a significance dichotomy (Section 3.2), the estimate assigns a probability of 0.85 to an increase of some magnitude, 0.48 to an increase of at least 30% and 0.035 to a doubling, so the series leans towards a modest increase while excluding increases of the magnitude projected early in the pandemic [1,2,3]. Second, no sustained increase in the post-onset slope was observed; this is a null result within a short series rather than evidence of stability. Third, SARS-CoV-2 infection was forensically ascertained in 24.3% of the suicide decedents of 2020–2021, 15.0% with ante-mortem laboratory confirmation, and these individuals were enriched for documented psychiatric history [9,10]; this finding is descriptive and provides information that is not available from civil death-registration data.

### 4.2. Comparison with the International ITS Evidence

Large multi-country ITS studies consistently reported no overall increase in suicide mortality during the early phases of the pandemic and, in several countries, documented declining rates [3,4,5,6,7,8]. The present study is consistent with that literature in its formal result: the pre-specified ITS model does not identify a significant change at onset. What distinguishes the present series is a numerical excess in the raw pandemic-period means that the model cannot resolve at this sample size, and it is worth setting out why resolution is not to be expected here. First, almost all of the international ITS literature analyses population-standardised rates; the present primary analysis is count-based, with a population-offset sensitivity model reported in Section 3.2 and Supplementary Table S2. Second, those studies cover national or supranational populations of tens of millions, within which heterogeneous regional trajectories are averaged; the present study covers a resident population of approximately 0.78 million, so that a single month of eight deaths rather than five is enough to move the estimate, and the confidence intervals are correspondingly wide. Third, an increase detectable in a two-county series need not be detectable, or present, at national scale. The appropriate reading of our result is therefore that a regional registry of this size cannot adjudicate effects of the magnitude at issue, not that it contradicts the aggregate picture.

### 4.3. Eastern European and Romanian Context

The comparison with countries of similar epidemiological and post-transition profile is more informative than the global aggregate. In Hungary, where suicide mortality had fallen by more than a third between 2010 and 2019, an interrupted time-series analysis found that the declining trend reversed during the pre-vaccination period of 2020, with an overall excess of 16.7% relative to the modelled expectation and larger excesses among men (18.5%) and in the 35–49 age group (32.8%) [30]; a subsequent analysis of the first two pandemic years found that the adverse effect attenuated by 2021 and disappeared among men [31]. In Poland, national police and registry data showed a rise in suicide among women across all age groups between 2019 and 2021 and an increase in attempts among the younger age bands, while male suicide did not increase [32]. Comparative work on “deaths of despair” across 27 European countries found that the pre-pandemic downward trend failed to continue in Poland and in the Baltic states during 2020–2021, in contrast to Southern Europe [33].

Our result—a non-significant level change at onset, with a raw pandemic-period excess of about 30% and no sustained trend change—is directionally compatible with this Central and Eastern European pattern but does not confirm it, since the interval around our estimate includes both the Hungarian order of magnitude and no effect. The regional commonality invoked in that literature is a population with historically high baseline suicide rates, a large rural share and thin specialist mental-health provision in which an acute systemic shock might produce a short-lived excess that subsequently attenuates; the present series is too small to test that hypothesis.

At national level, Romanian suicide mortality has declined over three decades, from approximately 13–14 per 100,000 in the early 2000s to about 9–10 per 100,000 by the end of the 2010s, placing the country close to the European Union average; the decline has been accompanied by a persistent male-to-female ratio of roughly 5:1 and a rural excess, with 57–60% of suicides occurring in rural settlements. Hanging predominates as the method, accounting for around three-quarters of cases nationally—a proportion exceeded in the present forensic series (93.9–97.1%), consistent with earlier Romanian county-level forensic studies [11]. The national trend figures are sourced from the WHO Global Health Observatory estimates as compiled by the World Bank [34]; the male-to-female ratio and rural excess are consistent with the national retrospective series reported by Dumitru et al. [35]. The distribution of suicide methods in the present registry was stable across periods: hanging accounted for 95.0% of deaths pre-pandemic (114/120), 93.6% during the pandemic (191/204) and 94.4% in the 2023–2024 supplementary period (67/71), with no other method exceeding a handful of cases in any period. We report this as an informative negative finding: the pandemic was not accompanied by a shift toward more or less lethal methods in this registry. The rural share among decedents (74.2%) markedly exceeds the rural share of the resident population of the two counties (approximately 45%, Section 4.4) and also exceeds the national rural share of suicides, a rural excess consistent in direction with the national pattern.

### 4.4. Characteristics of the Study Region

The generalisability of these findings depends on how the two counties compare with the rest of Romania, and the comparison is not neutral. Galați and Brăila lie in the Southeast development region, on the lower Danube. At the 2021 census, the resident population was 496,892 in Galați county and 281,452 in Brăila county [27]. Urbanisation differs between them—52.1% urban in Galați against 61.3% in Brăila—giving a combined urban share of approximately 55%, close to the national figure, so the region is not predominantly rural in the way that several other Romanian counties are. Both counties are, however, demographically older and shrinking faster than the country as a whole: the mean age of the resident population of Brăila county is 45.3 years against a national 42.4, its demographic ageing index is 170.7 elderly persons per 100 young persons against a national 121.2, and the county lost 12.4% of its residents between the 2011 and 2021 censuses; Galați county lost 7.3% over the same interval, with an ageing index of 131.1 [27]. Economically, the Southeast region has long ranked below the national average in gross domestic product per inhabitant, and both county seats are former heavy-industry and Danube port centres that have contracted substantially since 1990.

Two consequences follow. First, an older, contracting, economically peripheral population is plausibly more exposed to the isolating and economic effects of pandemic restrictions than the national average, consistent with the direction—though not the statistical significance—of our finding. Second, sustained depopulation means that a count-based series will understate any rate increase and overstate any rate decrease across the study window. As set out in Section 2.4, however, a smoothly log-linear denominator is absorbed by the linear trend term already in the model and therefore affects the intercept and the underlying trend rather than the estimated step at onset; the offset model reported in Section 3.2 confirms this. Findings from this region should be extrapolated to Romania as a whole only with these qualifications.

### 4.5. The Forensic Dimension

The forensic component represents the principal strength and novelty of the present study. Unlike civil mortality databases, forensic registries establish SARS-CoV-2 infection through medico-legal documentation and autopsy findings [9,10,14,15]. Whether the observed enrichment for psychiatric history reflects shared vulnerability, the psychological consequences of diagnosis and isolation, biological neuropsychiatric effects of infection, or differential ascertainment cannot be determined here, and the design does not permit any of these to be distinguished.

The value of autopsy-based ascertainment during the pandemic is not specific to suicide research. A body of forensic literature documented that post-mortem investigation was for a period the only means of establishing infection in decedents who had not been tested in life, that morphological findings alone are insufficiently specific for diagnosis, and that autopsy practice had to be reorganised around biosafety constraints and the risk posed by asymptomatic or undiagnosed infection—constraints that in several jurisdictions reduced autopsy volumes precisely when post-mortem evidence was most needed [10,36,37]. From a public-health perspective, the broader context also underscores the importance of maintaining suicide-prevention strategies and access to mental-health services during periods of major health-system disruption, consistent with the WHO LIVE LIFE framework [38]. This is the general form of the argument advanced here: where infection status is not established in life, only the medico-legal record can supply it, and civil registration cannot substitute.

The same period placed the health system itself under strain that plausibly bears on suicide risk. Healthcare workers experienced elevated infection and mortality across successive waves, and the reorganisation of services around pandemic care displaced other clinical activity, including specialist mental-health provision [39]. Analyses of medical liability and claims activity during the pandemic have been read as one indicator of that systemic pressure [40,41,42,43]. We note these as context for the mechanism by which a public-health emergency may raise suicide risk indirectly, through reduced access to care, rather than as findings of the present study.

### 4.6. Strengths and Limitations

Strengths of the study include exhaustive population-based case ascertainment under mandatory autopsy, integration of documented ante-mortem SARS-CoV-2 laboratory results with post-mortem morphological findings, an 84-month observation period, seasonal adjustment of the ITS model, and the use of a data-driven changepoint cross-check alongside the a priori model [19,20,21,22,23,24,25,26]. The pre-specified analytical hierarchy is reported and followed, and the full model output and monthly series are supplied so that every estimate can be reproduced independently. The study was designed and reported according to the STROBE recommendations [29].

The most consequential concerns the years 2023–2024. The observed decline to 2.96 deaths per month is too large and too abrupt to be epidemiologically plausible over two years, and it is best explained by incomplete ascertainment: a forensic case enters the registry only once the medico-legal report is finalised and the file closed, so the most recent years are systematically undercounted at the time of extraction. Two features support this reading rather than a genuine fall—the decline is monotonic across the two most recent years, which is the signature of a truncation process rather than of an epidemiological change, and no corresponding fall is recorded in national suicide statistics for the same period. For this reason, the primary analysis excludes 2023–2024; these years are presented only in the Supplementary Materials, and no conclusion of this paper depends on them. Definitive assessment requires re-extraction once registration is complete.

Second, and central to interpreting the primary result, the series is short and the population small. The primary model had 80% power to detect a level change of IRR ≥ 1.98 and approximately 19% power against an IRR of 1.30, so the study cannot adjudicate effects of the magnitude actually at issue in the pandemic suicide literature, which are typically in the range of ±10–30%. The non-significant primary result is therefore a statement about precision as much as about the phenomenon. Third, ascertainment of SARS-CoV-2 infection was limited by the available medico-legal documentation. Laboratory confirmation rested exclusively on documented ante-mortem RT-PCR results, as no post-mortem laboratory testing was performed; and 13 cases were classified as possible/probable solely on pulmonary findings morphologically compatible with COVID-19 at autopsy, which are not specific for SARS-CoV-2. Neither the 8.6% nor the 24.3% ascertainment prevalence should be interpreted as an estimate of the true prevalence of infection among suicide decedents; both are lower bounds constrained by testing practice. Systematic post-mortem virological testing, which was not available to Romanian forensic services during the reference period (Section 2.2), would be required to establish the true prevalence. Fourth, with 21 laboratory-confirmed and 13 possible/probable cases, the subgroup contrasts are hypothesis-generating; the doubled psychiatric-history proportion corresponds to a difference of a few individuals and is vulnerable to differential documentation, since decedents who had been in contact with clinical services during the pandemic are more likely both to have been tested and to have a recorded psychiatric history. Fifth, this ecological interrupted time-series analysis was restricted to a single region and lacked individual-level linkage to infection, vaccination, or migration databases, limiting causal inference and external generalisability. Finally, contemporaneous influences—socioeconomic support measures, healthcare reorganisation, alcohol availability, restrictions on movement—cannot be disentangled within a single ITS design, so any association observed in March 2020 would be attributable to the pandemic period as a whole and not to any specific mechanism [19,20,21,22].

### 4.7. Scientific and Applied Value of the Study

The imprecision of the primary estimate is stated without qualification in Section 4.1 and Section 4.6, and nothing in this section is intended to soften it. The contribution of the study does not, however, rest on the statistical significance of the level change at pandemic onset. It rests on five elements that are independent of that result.

First, the study shows that systematic identification of infection within the medico-legal investigation is feasible and yields usable epidemiological data. During 2020–2021, ascertainment of SARS-CoV-2 status, through ante-mortem laboratory documentation incorporated into the medico-legal file and through the pulmonary findings recorded at autopsy, formed part of routine forensic practice in this network, and it identified infection in a relevant proportion of suicide decedents (Section 3.5). This does not in itself justify permanent testing for SARS-CoV-2. What it supports is a standing medico-legal surveillance and testing capacity that can be activated and adapted rapidly to whichever infectious agent is epidemiologically relevant. Forensic medicine could thereby operate as a complementary component of epidemiological surveillance, particularly for violent, unexpected, or unexplained deaths, where the medico-legal examination can establish an infection status that conventional vital statistics do not capture. The SARS-CoV-2 experience is offered as a proof of concept for integrating the surveillance of emerging infections into the medico-legal infrastructure, not as a recommendation specific to one pathogen.

Second, the contribution is methodological. The study demonstrates that medico-legal ascertainment, interrupted time-series analysis with seasonal adjustment, and individual-level characterisation of infection can be combined within a single analytical framework. It supplies what is needed to repeat that framework elsewhere: the complete monthly series (Supplementary Table S3), the unedited model output (Supplementary Table S4) and a pre-specified analytical hierarchy that binds irrespective of the significance of the primary result (Section 2.4). The same pipeline can be applied without structural modification to other regional medico-legal registries in Romania and in European countries whose forensic systems share the mandatory-autopsy rule for violent death on which exhaustive ascertainment depends. The study thus proposes a reproducible framework for medico-legal surveillance during public-health emergencies and not only a local estimate of pandemic-period change; reproducibility of this kind is a precondition for multicentre work and for standardising future analyses across registries.

Third, the study is a regional contribution to an international literature in which longitudinal medico-legal data from Eastern Europe are markedly under-represented. The estimate of IRR = 1.29 must be read first in the light of its uncertainty and not as a demonstration of a 29% increase. Still, it is precisely that estimate, with its standard error of 0.244 on the log scale and the documented characteristics of the series, which can be entered into future pooled analyses. The international evidence on pandemic-period suicide mortality rests largely on national civil-registration series from particular countries and regions [4,5,6,7,8], and a fully characterised regional registry helps to narrow that geographical gap. In an individual-participant or time-series meta-analysis, the uncertainty of a single registry is combined with the information from other populations, so that the value of the present series does not depend solely on its power to detect an effect within one region. The series additionally offers what ecological studies built on civil mortality statistics rarely possess: individual-level medico-legal data and documented infection status for every decedent.

Fourth, the results bear on the preparedness of mental-health services for periods of crisis. From a public-health perspective, the findings support a balanced conclusion: the data do not support a large increase or a doubling of suicide mortality, but they do not exclude a modest increase (Section 3.2). That a modest increase cannot be excluded in a series of limited power must not be converted into a statement that an increase has been demonstrated. It is nevertheless relevant to mental-health contingency planning: during future periods of social restriction, epidemics or other major disruptions of community life, mental-health services may require monitoring mechanisms and additional capacity even in the absence of evidence that suicide mortality will rise sharply. This study provides a regional surveillance and preparedness signal; it provides neither a clinical recommendation nor evidence of a causal relationship between the pandemic and suicide.

Fifth, and structurally the most important, the study demonstrates the added value of medico-legal registries over conventional mortality statistics. Civil death registration records the fact and the cause of death; it does not ordinarily record, at the same level of detail, the circumstances of the death, documented psychiatric history, the results of infection testing, or the other individual characteristics reported in Table 5. The medico-legal registry can therefore serve as a complementary source for population surveillance, supporting interoperability between medico-legal registries, public-health systems, and vital statistics, and enabling faster, more detailed monitoring during future emergencies. Seen in this light, the study does not only reach a conclusion about suicide and COVID-19 in one region: it demonstrates the value of the medico-legal infrastructure as a platform for epidemiological surveillance, capable of informing both population-level mortality trends and the individual characteristics of the deceased.

## 5. Conclusions

In a complete southeastern Romanian forensic suicide registry, the pre-specified, seasonally adjusted segmented Poisson regression did not demonstrate a statistically significant change in suicide mortality at the onset of the COVID-19 pandemic (level IRR = 1.29, 95% CI 0.80–2.08, *p* = 0.302), and no sustained change in slope thereafter. The raw monthly means were higher during the pandemic period than before it (6.00 vs. 4.62; unadjusted rate ratio 1.30, 95% CI 1.04–1.63), but this unadjusted comparison does not account for temporal trend, seasonality or serial correlation and should therefore be interpreted descriptively. Because the study had 80% power only against a level change of about a doubling, the correct conclusion is that this registry is compatible with a modest pandemic-period increase and equally compatible with none, and not that no increase occurred. In posterior terms, the data assign 85% probability to an increase of some magnitude and 3.5% to a doubling (Section 3.2). Forensic ascertainment identified documented ante-mortem laboratory-confirmed SARS-CoV-2 infection or compatible post-mortem pulmonary findings in 24.3% of the suicide decedents of 2020–2021 (15.0% laboratory-confirmed); this descriptive observation, which civil death registration could not have produced, is the study’s principal positive contribution, and it should not be read as an estimate of the true prevalence of infection among suicide decedents.

Three directions follow for research. First, the registry should be extended to the neighbouring counties of the Southeast region and the analysis repeated on population-standardised rates, which would raise the catchment population to a scale at which regional estimates become stable. Second, the series should be re-extracted once registration of 2023–2024 cases is complete, which is the only way to establish whether the apparent post-2022 decline is real. Third, where legally and ethically feasible, linking forensic records to national infection, vaccination, and prescription databases would move the infection–suicide question from ecological association toward individual-level inference and allow the confounding by documentation intensity identified above to be addressed directly. Future studies would benefit from systematic post-mortem virological testing maintained throughout the study period, allowing laboratory confirmation independent of ante-mortem testing history.

For prevention and preparedness, two implications follow and should be kept distinct. A regional forensic registry covering under a million residents cannot resolve pandemic-related changes in suicide mortality of the magnitude reported elsewhere, and prevention policy should not be founded on regional series of this size; the result is a surveillance and preparedness signal, not a causal finding and not a clinical recommendation. What such registries can contribute, and what conventional mortality statistics cannot, is individual-level infection status and circumstantial detail in decedents; this argues for a standing medico-legal surveillance capacity, activated and adapted to the relevant agent in future epidemics, and for interoperability between medico-legal registries, public-health systems and vital statistics (Section 4.7). Mental-health contingency planning for future public-health emergencies should continue to be directed first at populations with the profile identified here, older, rural or peripheral, with pre-existing psychiatric contact and limited access to specialist services, on the strength of the wider evidence base rather than of this series alone, while retaining the monitoring capacity needed to detect a modest increase that a series of this size cannot exclude.

## Figures and Tables

**Table 1:** Annual and period suicide counts, Galați–Brăila, 2018–2024 (*n* = 395; 84 months). The pre-pandemic/pandemic partition uses March 2020 (national state of emergency) as the time origin; 2023–2024 is reported only as a supplementary period.

Period/Year	Deaths	Deaths per Month
2018	58	4.83
2019	55	4.58
2020	65	5.42
2021	75	6.25
2022	71	5.92
2023 (supplementary)	42	3.50
2024 (supplementary)	29	2.42
Pre-pandemic (January 2018–February 2020)	120	4.62
Pandemic (March 2020–December 2022)	204	6.00
Supplementary (January 2023–December 2024)	71	2.96

**Table 2:** Seasonally adjusted segmented Poisson regression of monthly suicide counts, restricted series January 2018–December 2022 (*n* = 60 months). Time origin = March 2020. IRR = incidence rate ratio. Pearson dispersion = 0.80 (no evidence of overdispersion). No IRR is reported for the seasonal harmonic terms because a sine or cosine term of an annual harmonic pair has no standalone rate-ratio interpretation: the two terms jointly parameterise a single seasonal curve, and the exponentiated coefficient of either one in isolation does not correspond to a contrast between meaningful exposure levels. The seasonal effect is therefore reported as the amplitude and phase of the fitted harmonic pair in the text.

Term	Coef. (β)	IRR	95% CI	*p*
Intercept	1.573	—	—	<0.001
Time (per month)	−0.004	0.996	0.972–1.021	0.776
Level change (onset)	0.252	1.287	0.797–2.076	0.302
Slope change (post-onset)	0.007	1.007	0.979–1.035	0.639
Seasonal sine	0.075	n.a.	n.a.	0.352
Seasonal cosine	−0.161	n.a.	n.a.	0.046

Note: n.a., not applicable.

**Table 3:** Descriptive, unadjusted period rate ratio for monthly suicide counts, with the two-rate Poisson 95% confidence interval. This comparison is not the primary analysis; it does not adjust for the underlying time trend, seasonality, or serial correlation, and it is reported for descriptive purposes only (Section 2.6 and Section 3.3). Comparisons involving the supplementary period 2023–2024 are given in Supplementary Table S1.

Comparison	Rate (per Month)	IRR	95% CI
Pandemic vs. pre-pandemic	6.00 vs. 4.62	1.30	1.04–1.63

**Table 4:** Structural breaks from changepoint detection versus the a priori pandemic onset, with segment-mean levels. The penalty used for PELT is stated in Section 2.5.

Method	Detected Break(s)	Segment Level (Deaths/Month)
Binary segmentation (2 breaks)	February 2020; January 2023	January 2018–January 2020: 4.52
PELT (penalty as reported)	January 2023 only	February 2020–December 2022: 6.03
A-priori ITS onset	March 2020	January 2023–December 2024: 2.96 (supplementary)

**Table 5:** Profile of suicide decedents by SARS-CoV-2 ascertainment tier. Laboratory-confirmed cases were based on documented ante-mortem RT-PCR results; possible/probable cases were based on pulmonary findings morphologically compatible with COVID-19 at autopsy in the absence of laboratory confirmation. The two tiers are shown separately because they carry different evidential weight. All contrasts are descriptive; no significance test was performed.

Variable	Laboratory-Confirmed (*n* = 21)	Possible/Probable (*n* = 13)	All Ascertained (*n* = 34)	Not Ascertained (*n* = 361)
Mean age (years)	57.1 (SD 14.0)	52.5 (SD 18.9)	55.4 (SD 15.9)	54.0 (SD 16.3)
Male sex	90.5%	92.3%	91.2%	86.7%
Rural residence	52.4%	76.9%	61.8%	74.2%
Hanging (method)	100.0%	92.3%	97.1%	93.9%
Documented psychiatric history	57.1% (12/21)	61.5% (8/13)	58.8% (20/34)	29.4% (106/361)
Recorded psychiatric diagnosis categories	Depressive disorder 11 (52.4%); alcohol dependence 1 (4.8%)	Depressive disorder 5 (38.5%); alcohol dependence 2 (15.4%); dementia 1 (7.7%)	Depressive disorder 16 (47.1%); alcohol dependence 3 (8.8%); dementia 1 (2.9%)	Depressive disorder 66 (18.3%); alcohol dependence 16 (4.4%); personality disorder 11 (3.0%); schizophrenia 6 (1.7%); dementia 5 (1.4%); bipolar disorder 2 (0.6%)

## Data Availability

The monthly suicide count series analysed in this study, the resident-population denominator series, and the full software output of every model reported are provided as Supplementary Materials. The underlying individual-level medico-legal records cannot be released; anonymised record-level data are available from the corresponding author on reasonable request, subject to forensic data-governance approval. The analysis scripts that produce the model output, the changepoint runs and the posterior summaries of Supplementary Table S6 are available from the corresponding author on request.

## Supplementary Materials

The following supporting information can be downloaded at: https://www.mdpi.com/article/10.3390/diagnostics16172736/s1. Figure S1: Observed monthly series and fitted primary model—observed monthly suicide counts over the full 84 months, with the supplementary period distinguished and the fitted primary model with its 95% confidence band superimposed. Table S1: Period rate ratios, including the supplementary period 2023–2024, with the ascertainment caveat. Table S2: Sensitivity analyses—negative-binomial specification, series truncated in December 2023, and the population-offset model of Section 2.4, each with full coefficient tables. Table S3: Monthly suicide count series and resident-population denominator, January 2018–December 2024, with the 60-month primary analysis window identified. Table S4: Unedited software output of every model reported, including the changepoint runs on the 84-month and 60-month series. Table S5: Residual diagnostics of the primary model, including the Durbin–Watson statistic and Ljung–Box tests at lags 1–12. Table S6: Posterior summaries for the level change at pandemic onset, including posterior probabilities, posterior median, credible intervals, and the software output of the computation.
