# Supplementary material for: COVID-19 and Suicide Mortality in a Population-Based Medico-Legal Registry: An Interrupted Time-Series and Changepoint Analysis from Southeast Romania
Source: Diagnostics (Basel). 2026 Aug 26;16(17):2736. doi: 10.3390/diagnostics16172736 (PMC13564694; doi:10.3390/diagnostics16172736)
Supplement: Supplementary file 1 [file diagnostics-16-02736-s001.zip › diagnostics-4468582-supplementary.pdf]

Supplementary Materials

COVID-19 and Suicide Mortality in a Population-Based Medico-Legal Registry: An Interrupted Time-Series and Changepoint Analysis from South-East Romania

manuscript ID 4468582

This file contains Supplementary Tables S1-S6 and Supplementary Figure S1. Table S3 gives the complete monthly count series from which every estimate reported in the manuscript is derived, and Table S4 reproduces the unedited output of every model fitted, exactly as the software produced it, so that each figure in Tables 2-4 of the manuscript can be checked independently. Table S6 expresses the level change of the primary model as a posterior distribution (Section 3.2 of the manuscript), computed from the fitted coefficients reported in Table S4.

Table S1. Period rate ratios, including the supplementary period 2023-2024

Descriptive, unadjusted two-rate Poisson comparisons of mean monthly counts. Each comparison assumes a constant rate within each period and makes no allowance for the underlying time trend, for seasonality or for serial correlation; the confidence interval is $\exp(\log RR \pm 1.96 \cdot \sqrt{(1/D_1 + 1/D_2)})$ and reflects sampling variation in the two death totals alone. None of these comparisons is the primary analysis and no substantive conclusion of the paper rests on any of them (Sections 2.6, 3.3 and 4.6).

Table S1a. Period descriptives

Period	Months	Deaths	Deaths per month
Pre-pandemic (Jan 2018 - Feb 2020)	26	120	4.62
Pandemic (Mar 2020 - Dec 2022)	34	204	6.00
Supplementary (Jan 2023 - Dec 2024)	24	71	2.96
**Whole series (Jan 2018 - Dec 2024)**	**84**	**395**	**4.70**

Table S1b. Unadjusted period rate ratios

Comparison	Rates (per month)	IRR	95% CI	p
Pandemic vs pre-pandemic	6.00 vs 4.62	1.300	1.038 - 1.629	0.023
Supplementary vs pre-pandemic	2.96 vs 4.62	0.641	0.478 - 0.860	0.003
Supplementary vs pandemic	2.96 vs 6.00	0.493	0.376 - 0.646	<0.001

Caveat applying to both rows involving the supplementary period: case ascertainment for 2023-2024 is demonstrably incomplete at the date of data extraction, because a forensic case enters the registry only once the medico-legal report is finalised and the file is closed. The apparent halving of the monthly rate after 2022 is attributed to that truncation and not to a fall in suicide mortality (Section 4.6). These two comparisons are reported for completeness only and must not be read as epidemiological findings.

Table S2. Sensitivity analyses

All three models use the same specification as the primary model of Table 2: $\log(\mu_t) = \beta_0 + \beta_1 \cdot t + \beta_2 \cdot D_t + \beta_3 \cdot (t - t_0) \cdot D_t + \beta_4 \cdot \sin(2\pi m/12) + \beta_5 \cdot \cos(2\pi m/12)$, with $t_0 = 27$ (March 2020). β_2 is the level change at onset and β_3 the

post-onset slope change. No IRR is reported for the seasonal harmonic terms, for the reason given in the legend of Table 2.

Table S2a. Population-offset model, restricted series (Jan 2018 - Dec 2022, n = 60)

Offset = log of the monthly resident population of Galati and Braila counties combined, constructed by log-linear interpolation between the two national census totals (857,379 in October 2011; 778,344 in December 2021) and reported in full in Table S3. The intercept is on the log-rate scale and is therefore not comparable with the intercept of the count-based model.

Term	Coef. (β)	IRR	95% CI	p
Intercept	−12.030	—	—	<0.001
Time (per month)	−0.003	0.997	0.973 - 1.022	0.825
**Level change (onset)**	**0.252**	**1.287**	**0.797 - 2.076**	**0.302**
Slope change (post-onset)	0.007	1.007	0.979 - 1.035	0.639
Seasonal sine	0.075	n.a.	n.a.	0.352
Seasonal cosine	−0.161	n.a.	n.a.	0.046

The level change is identical to that of the count-based primary model (IRR = 1.287, 95% CI 0.797-2.076, p = 0.302), as are the slope change and both seasonal terms; only the intercept and the linear trend differ. This is the expected result: over the five-year analysis window the resident population declines close to log-linearly, so a log-linear denominator entered as an offset is absorbed by the intercept and the trend term and leaves the level and slope coefficients unchanged (Section 2.4).

Table S2b. Negative-binomial specification, restricted series (Jan 2018 - Dec 2022, n = 60)

Dispersion parameter α estimated by maximum likelihood.

Term	Coef. (β)	IRR	95% CI	p
Intercept	1.573	—	—	<0.001
Time (per month)	−0.004	0.996	0.972 - 1.021	0.776
**Level change (onset)**	**0.252**	**1.287**	**0.797 - 2.076**	**0.302**
Slope change (post-onset)	0.007	1.007	0.979 - 1.035	0.639
Seasonal sine	0.075	n.a.	n.a.	0.352
Seasonal cosine	−0.161	n.a.	n.a.	0.046
**Dispersion α**	5.6×10^{-8}	—	—	0.999

The negative-binomial fit returns coefficients numerically identical to the Poisson fit, with the dispersion parameter estimated at essentially zero. This is the expected degenerate outcome when the data show no overdispersion beyond that already accommodated by the Poisson model, and it is consistent with the Pearson dispersion statistic of 0.80 reported in Table S5.

Table S2c. Poisson model on the series truncated at December 2023 (n = 72)

Term	Coef. (β)	IRR	95% CI	p
Intercept	1.586	—	—	<0.001
Time (per month)	−0.004	0.996	0.972 - 1.020	0.726
**Level change (onset)**	**0.433**	**1.542**	**0.980 - 2.427**	**0.061**
Slope change (post-onset)	−0.007	0.993	0.968 - 1.020	0.621

Term	Coef. (β)	IRR	95% CI	p
Seasonal sine	0.057	n.a.	n.a.	0.448
Seasonal cosine	−0.137	n.a.	n.a.	0.070

This model is reported because counts for the most recent complete year are the most exposed to registration delay. The level change moves from IRR 1.29 on the 60-month window to IRR 1.54 on the 72-month window and to IRR 1.67 on the full 84-month window reported at first submission: the more of the incompletely registered post-2022 period is retained, the larger the apparent step at onset becomes. That gradient is itself evidence that the 84-month estimate was driven by the ascertainment artefact rather than by the pandemic, and it is the reason the 60-month window is the one used for the primary analysis.

Table S3. Monthly suicide count series and resident-population denominator, January 2018 - December 2024

Complete monthly series of the medico-legal suicide registry of Galati and Braila counties (N = 395 over 84 months). The denominator column is the resident population of the two counties combined, obtained by log-linear interpolation between the census totals of 857,379 (October 2011) and 778,344 (December 2021) and continued at the same monthly log-slope of −0.00079 (−0.95% per year) beyond December 2021; it is the offset series used in Table S2a. The 60-month primary analysis window is January 2018 - December 2022; the months of 2023 and 2024 form the supplementary period and are excluded from the primary analysis. Annual totals: 58 (2018), 55 (2019), 65 (2020), 75 (2021), 71 (2022), 42 (2023), 29 (2024). Period totals: 120 pre-pandemic (Jan 2018 - Feb 2020), 204 pandemic (Mar 2020 - Dec 2022), 71 supplementary (Jan 2023 - Dec 2024).

Month	Deaths	Population	Window	Month	Deaths	Population	Window
2018-01	9	807,890	Pre-pand.	2021-07	8	781,435	Pandemic
2018-02	3	807,250	Pre-pand.	2021-08	7	780,816	Pandemic
2018-03	4	806,610	Pre-pand.	2021-09	2	780,197	Pandemic
2018-04	7	805,971	Pre-pand.	2021-10	5	779,579	Pandemic
2018-05	6	805,333	Pre-pand.	2021-11	7	778,961	Pandemic
2018-06	5	804,694	Pre-pand.	2021-12	5	778,344	Pandemic
2018-07	3	804,057	Pre-pand.	2022-01	3	777,727	Pandemic
2018-08	3	803,420	Pre-pand.	2022-02	7	777,111	Pandemic
2018-09	3	802,783	Pre-pand.	2022-03	6	776,495	Pandemic
2018-10	5	802,147	Pre-pand.	2022-04	3	775,880	Pandemic
2018-11	5	801,511	Pre-pand.	2022-05	8	775,265	Pandemic
2018-12	5	800,876	Pre-pand.	2022-06	6	774,651	Pandemic
2019-01	2	800,242	Pre-pand.	2022-07	7	774,037	Pandemic
2019-02	5	799,607	Pre-pand.	2022-08	7	773,424	Pandemic
2019-03	4	798,974	Pre-pand.	2022-09	6	772,811	Pandemic
2019-04	5	798,341	Pre-pand.	2022-10	7	772,198	Pandemic
2019-05	6	797,708	Pre-pand.	2022-11	6	771,586	Pandemic
2019-06	4	797,076	Pre-pand.	2022-12	5	770,975	Pandemic
2019-07	6	796,444	Pre-pand.	2023-01	4	770,364	Suppl.
2019-08	5	795,813	Pre-pand.	2023-02	3	769,754	Suppl.
2019-09	2	795,183	Pre-pand.	2023-03	6	769,144	Suppl.
2019-10	6	794,553	Pre-pand.	2023-04	3	768,534	Suppl.

Month	Deaths	Population	Window	Month	Deaths	Population	Window
2019-11	6	793,923	Pre-pand.	2023-05	3	767,925	Suppl.
2019-12	4	793,294	Pre-pand.	2023-06	8	767,317	Suppl.
2020-01	0	792,665	Pre-pand.	2023-07	0	766,709	Suppl.
2020-02	7	792,037	Pre-pand.	2023-08	2	766,101	Suppl.
2020-03	5	791,410	Pandemic	2023-09	4	765,494	Suppl.
2020-04	9	790,782	Pandemic	2023-10	6	764,888	Suppl.
2020-05	5	790,156	Pandemic	2023-11	1	764,281	Suppl.
2020-06	11	789,530	Pandemic	2023-12	2	763,676	Suppl.
2020-07	3	788,904	Pandemic	2024-01	0	763,071	Suppl.
2020-08	5	788,279	Pandemic	2024-02	1	762,466	Suppl.
2020-09	5	787,654	Pandemic	2024-03	3	761,862	Suppl.
2020-10	5	787,030	Pandemic	2024-04	6	761,258	Suppl.
2020-11	4	786,407	Pandemic	2024-05	7	760,655	Suppl.
2020-12	6	785,783	Pandemic	2024-06	1	760,052	Suppl.
2021-01	4	785,161	Pandemic	2024-07	3	759,450	Suppl.
2021-02	1	784,539	Pandemic	2024-08	2	758,848	Suppl.
2021-03	8	783,917	Pandemic	2024-09	0	758,247	Suppl.
2021-04	11	783,296	Pandemic	2024-10	2	757,646	Suppl.
2021-05	9	782,675	Pandemic	2024-11	3	757,046	Suppl.
2021-06	8	782,055	Pandemic	2024-12	1	756,446	Suppl.

Table S4. Unedited software output of every model reported

The complete console output of the analysis - the generalised-linear-model summaries of the primary model, the negative-binomial model, the model truncated at December 2023 and the population-offset model, the power calculation, the residual diagnostics, the period rate ratios and the full penalty sweep of the changepoint runs on both the 84-month and the 60-month series - is reproduced below exactly as the software produced it. Nothing has been edited, reformatted or abridged.

```
==============================================================================
PRIMARY MODEL - segmented Poisson, Jan 2018 - Dec 2022 (n = 60 months), time origin t0 = 27 (March
2020)  [Table 2]
==============================================================================
                 Generalized Linear Model Regression Results
==============================================================================
Dep. Variable:                  count   No. Observations:                   60
Model:                            GLM   Df Residuals:                       54
Model Family:                 Poisson   Df Model:                            5
Link Function:                    Log   Scale:                          1.0000
Method:                          IRLS   Log-Likelihood:                -127.68
Date:                Fri, 14 Aug 2026   Deviance:                       49.324
Time:                        05:36:59   Pearson chi2:                     43.3
No. Iterations:                     4   Pseudo R-squ. (CS):             0.1606
Covariance Type:            nonrobust
==============================================================================
                 coef    std err          z      P>|z|      [0.025      0.975]
------------------------------------------------------------------------------
const          1.5732      0.188      8.368      0.000       1.205       1.942
t             -0.0036      0.012     -0.285      0.776      -0.028       0.021
D              0.2520      0.244      1.032      0.302      -0.226       0.730
Dt             0.0067      0.014      0.469      0.639      -0.021       0.035
sin1           0.0749      0.081      0.930      0.352      -0.083       0.233
cos1          -0.1605      0.081     -1.994      0.046      -0.318      -0.003
==============================================================================

                     Term   Coef    IRR CI_low CI_high     p
                Intercept  1.573      -      -       - 0.000
         Time (per month) -0.004  0.996  0.972   1.021 0.776
     Level change (onset)  0.252  1.287  0.797   2.076 0.302
Slope change (post-onset)  0.007  1.007  0.979   1.035 0.639
            Seasonal sine  0.075   n.a.   n.a.    n.a. 0.352
          Seasonal cosine -0.161   n.a.   n.a.    n.a. 0.046

Pearson dispersion = 0.80
Annual harmonic: amplitude = 0.177 on the log scale, peak at month 5.2

==============================================================================
POWER  [Section 3.2]
==============================================================================
SE(log level change) = 0.244
Detectable at 80% power, alpha = 0.05 (two-sided): IRR >= 1.98 or <= 0.50
Power against a true IRR of 1.30: 18.9%

==============================================================================
RESIDUAL DIAGNOSTICS  [Table S5]
==============================================================================
Pearson dispersion = 0.80
Durbin-Watson = 2.20
    lb_stat  lb_pvalue
1     1.592      0.207
2     2.920      0.232
3     3.150      0.369
4     4.460      0.347
5     9.611      0.087
6     9.872      0.130
7    11.180      0.131
8    11.913      0.155
9    15.260      0.084
```

```
10   15.286      0.122
11   15.737      0.151
12   16.435      0.172

==============================================================================
SENSITIVITY 1 - negative binomial, restricted series  [Table S2]
==============================================================================
                     NegativeBinomial Regression Results
==============================================================================
Dep. Variable:                  count   No. Observations:                   60
Model:               NegativeBinomial   Df Residuals:                       54
Method:                           MLE   Df Model:                            5
Date:                Fri, 14 Aug 2026   Pseudo R-squ.:                     nan
Time:                        05:37:01   Log-Likelihood:                -127.68
converged:                      False   LL-Null:                           nan
Covariance Type:            nonrobust   LLR p-value:                       nan
==============================================================================
                 coef    std err          z      P>|z|      [0.025      0.975]
------------------------------------------------------------------------------
const          1.5732      0.188      8.368      0.000       1.205       1.942
t             -0.0036      0.012     -0.285      0.776      -0.028       0.021
D              0.2519      0.244      1.032      0.302      -0.226       0.730
Dt             0.0067      0.014      0.469      0.639      -0.021       0.035
sin1           0.0749      0.081      0.930      0.352      -0.083       0.233
cos1          -0.1605      0.081     -1.994      0.046      -0.318      -0.003
alpha       5.626e-08   3.68e-05      0.002      0.999   -7.21e-05    7.22e-05
==============================================================================

Estimated dispersion parameter alpha = 0.000000

                     Term   Coef    IRR CI_low CI_high     p
                Intercept  1.573      -      -       - 0.000
        Time (per month) -0.004  0.996  0.972   1.021 0.776
    Level change (onset)  0.252  1.287  0.797   2.076 0.302
Slope change (post-onset)  0.007  1.007  0.979   1.035 0.639
            Seasonal sine  0.075   n.a.   n.a.    n.a. 0.352
          Seasonal cosine -0.161   n.a.   n.a.    n.a. 0.046

==============================================================================
SENSITIVITY 2 - Poisson, series truncated at December 2023  [Table S2]
==============================================================================
                 Generalized Linear Model Regression Results
==============================================================================
Dep. Variable:                  count   No. Observations:                   72
Model:                            GLM   Df Residuals:                       66
Model Family:                 Poisson   Df Model:                            5
Link Function:                    Log   Scale:                          1.0000
Method:                          IRLS   Log-Likelihood:                -156.83
Date:                Fri, 14 Aug 2026   Deviance:                       73.635
Time:                        05:37:01   Pearson chi2:                     62.5
No. Iterations:                     4   Pseudo R-squ. (CS):             0.1599
Covariance Type:            nonrobust
==============================================================================
                 coef    std err          z      P>|z|      [0.025      0.975]
------------------------------------------------------------------------------
const          1.5857      0.187      8.467      0.000       1.219       1.953
t             -0.0044      0.012     -0.350      0.726      -0.029       0.020
D              0.4332      0.231      1.873      0.061      -0.020       0.887
Dt            -0.0066      0.013     -0.495      0.621      -0.033       0.019
sin1           0.0569      0.075      0.758      0.448      -0.090       0.204
cos1          -0.1369      0.076     -1.811      0.070      -0.285       0.011
==============================================================================

                     Term   Coef    IRR CI_low CI_high     p
                Intercept  1.586      -      -       - 0.000
        Time (per month) -0.004  0.996  0.972    1.02 0.726
    Level change (onset)  0.433  1.542   0.98   2.427 0.061
Slope change (post-onset) -0.007  0.993  0.968    1.02 0.621
            Seasonal sine  0.057   n.a.   n.a.    n.a. 0.448
          Seasonal cosine -0.137   n.a.   n.a.    n.a. 0.070
```

```
==============================================================================
SENSITIVITY 3 - population-offset model, restricted series  [Table S2]
==============================================================================
Log-linear interpolation between census totals 857379 (2011-10) and 778344 (2021-12):
monthly log-slope b = -0.0007927  (-0.95% per year)
                 Generalized Linear Model Regression Results
==============================================================================
Dep. Variable:                  count   No. Observations:                   60
Model:                            GLM   Df Residuals:                       54
Model Family:                 Poisson   Df Model:                            5
Link Function:                    Log   Scale:                          1.0000
Method:                          IRLS   Log-Likelihood:                -127.68
Date:                Fri, 14 Aug 2026   Deviance:                       49.324
Time:                        05:37:01   Pearson chi2:                     43.3
No. Iterations:                     4   Pseudo R-squ. (CS):             0.1737
Covariance Type:            nonrobust
==============================================================================
                 coef    std err          z      P>|z|      [0.025      0.975]
------------------------------------------------------------------------------
const        -12.0297      0.188    -63.987      0.000     -12.398     -11.661
t             -0.0028      0.012     -0.221      0.825      -0.027       0.022
D              0.2520      0.244      1.032      0.302      -0.226       0.730
Dt             0.0067      0.014      0.469      0.639      -0.021       0.035
sin1           0.0749      0.081      0.930      0.352      -0.083       0.233
cos1          -0.1605      0.081     -1.994      0.046      -0.318      -0.003
==============================================================================

                     Term    Coef    IRR CI_low CI_high     p
                Intercept -12.030      -      -       - 0.000
         Time (per month)  -0.003  0.997  0.973   1.022 0.825
     Level change (onset)   0.252  1.287  0.797   2.076 0.302
Slope change (post-onset)   0.007  1.007  0.979   1.035 0.639
            Seasonal sine   0.075   n.a.   n.a.    n.a. 0.352
          Seasonal cosine  -0.161   n.a.   n.a.    n.a. 0.046

Level change, count model  : IRR = 1.287 (0.797-2.076), p = 0.302
Level change, offset model : IRR = 1.287 (0.797-2.076), p = 0.302


==============================================================================
PERIOD RATE RATIOS  [Tables 3 and S1]
==============================================================================
                           Period  Months  Deaths  Deaths per month
 Pre-pandemic (Jan 2018-Feb 2020)      26     120              4.62
     Pandemic (Mar 2020-Dec 2022)      34     204              6.00
Supplementary (Jan 2023-Dec 2024)      24      71              2.96

                   Comparison   IRR  CI_low  CI_high      p
     Pandemic vs Pre-pandemic 1.300   1.038    1.629 0.0226
Supplementary vs Pre-pandemic 0.641   0.478    0.860 0.0030
    Supplementary vs Pandemic 0.493   0.376    0.646 0.0000


==============================================================================
CHANGEPOINT DETECTION  [Table 4]
==============================================================================
[84-month series] n = 84, sample variance = 6.07, PELT penalty = 3*Var*log(n) = 80.6
    PELT  pen x0.5  -> 2023-01
    PELT  pen x1    -> 2023-01
    PELT  pen x2    -> none
    PELT  pen x4    -> none
    PELT  pen x8    -> none
    BinSeg 1 bkp(s) -> 2023-01 | segment means: 5.40, 2.96
    BinSeg 2 bkp(s) -> 2020-02, 2023-01 | segment means: 4.52, 6.03, 2.96
[60-month restricted series] n = 60, sample variance = 4.84, PELT penalty = 3*Var*log(n) = 59.4
    PELT  pen x0.5  -> 2020-02
    PELT  pen x1    -> none
    PELT  pen x2    -> none
    PELT  pen x4    -> none
    PELT  pen x8    -> none
    BinSeg 1 bkp(s) -> 2020-02 | segment means: 4.52, 6.03
    BinSeg 2 bkp(s) -> 2020-02, 2020-07 | segment means: 4.52, 7.40, 5.80
```

```
Figure S1 written to output/figureS1_observed_vs_fitted.png
```

Table S5. Residual diagnostics of the primary model

Because the validity of the Wald confidence interval for the level change depends on the absence of residual serial correlation, the Pearson residuals of the primary model were examined for autocorrelation. The Durbin-Watson statistic is close to the value of 2 expected under no autocorrelation, and the Ljung-Box test detects no significant autocorrelation at any lag from 1 to 12. The Wald interval reported in Table 2 is therefore not compromised by residual serial correlation.

Diagnostic	Value	Interpretation
Pearson dispersion (χ^2/df)	0.80	No overdispersion
Durbin-Watson	2.20	No first-order autocorrelation
Ljung-Box, lags 1-12	all $p \geq 0.084$	No autocorrelation at any lag

Table S5b. Ljung-Box test, lag by lag

Lag	Ljung-Box statistic	p
1	1.592	0.207
2	2.920	0.232
3	3.150	0.369
4	4.460	0.347
5	9.611	0.087
6	9.872	0.130
7	11.180	0.131
8	11.913	0.155
9	15.260	0.084
10	15.286	0.122
11	15.736	0.151
12	16.435	0.172

Table S6. Posterior summaries for the level change at pandemic onset

The primary model returns a Wald estimate of the level change at pandemic onset ($\beta_2 = 0.252$, SE = 0.244; IRR = 1.29, 95% CI 0.80 - 2.08, p = 0.302; Table 2 and Table S4). Because a dichotomised significance statement conveys little about a series of this length, the same information is expressed here as a posterior distribution for the level change under a flat (improper uniform) prior on the log scale, $\log(\mathrm{IRR}) \mid \mathrm{data} \sim \mathrm{Normal}(\beta_2, \mathrm{SE}^2)$. Under this prior the posterior is the normalised likelihood, so that the posterior median equals the point estimate and the 95% credible interval coincides numerically with the Wald confidence interval; the added information lies in the tail probabilities, which state directly how compatible the data are with increases of a given size. The same computation is reported for the sensitivity model truncated at December 2023 (Table S2c). All figures were computed from the fitted statsmodels coefficients, not from the rounded published values; the computation is reproduced in Table S6b exactly as the software produced it.

Quantity	Primary model (Jan 2018 - Dec 2022, n = 60)	Sensitivity model truncated at Dec 2023 (n = 72)
Level-change coefficient β_2 (SE), log scale	0.252 (0.244)	0.433 (0.231)

Quantity	Primary model (Jan 2018 - Dec 2022, n = 60)	Sensitivity model truncated at Dec 2023 (n = 72)
Posterior median IRR	1.29	1.54
**P(IRR > 1.0) - any increase**	**0.849**	**0.969**
P(IRR > 1.2)	0.612	0.861
P(IRR > 1.3) - magnitude of the raw period means	0.483	0.770
P(IRR > 1.5)	0.265	0.548
**P(IRR > 2.0) - a doubling**	**0.035**	**0.131**
P(IRR < 1.0) - any decrease	0.151	0.031
90% credible interval for IRR	0.86 - 1.92	1.05 - 2.26
95% credible interval for IRR	0.80 - 2.08	0.98 - 2.43

Reading of the table: the data give a posterior probability of 0.85 to an increase of some size and of 0.035 to a doubling; they are close to indeterminate (0.48) as to whether the increase reached the 30% implied by the raw period means. On the truncated series, which retains part of the incompletely registered post-2022 period and is reported for sensitivity only, the probabilities are higher throughout (Section 4.6 of the manuscript explains why retaining those months inflates the apparent step at onset).

Table S6b. Software output of the posterior computation

```
==============================================================================
POSTERIOR SUMMARIES FOR THE LEVEL CHANGE AT PANDEMIC ONSET  [Table S6]
==============================================================================
statsmodels 0.14.6; series source: Table S3 (embedded)

PRIMARY MODEL - segmented Poisson, Jan 2018 - Dec 2022 (n = 60)  [Table 2]
  level-change coefficient beta_D = 0.2520   SE = 0.2441   IRR = 1.287 (0.797-2.076), p = 0.302
  posterior: log(IRR) ~ N(0.2520, 0.2441^2)  [flat prior on the log scale]
  P(IRR > 1.0) = 0.8491
  P(IRR > 1.2) = 0.6124
  P(IRR > 1.3) = 0.4831
  P(IRR > 1.5) = 0.2648
  P(IRR > 2.0) = 0.0354
  P(IRR < 1.0) = 0.1509
  posterior median IRR = 1.287
  90% credible interval = 0.861 - 1.922
  95% credible interval = 0.797 - 2.076

SENSITIVITY - Poisson, series truncated at Dec 2023 (n = 72)  [Table S2c]
  level-change coefficient beta_D = 0.4332   SE = 0.2313   IRR = 1.542 (0.980-2.427), p = 0.061
  posterior: log(IRR) ~ N(0.4332, 0.2313^2)  [flat prior on the log scale]
  P(IRR > 1.0) = 0.9694
  P(IRR > 1.2) = 0.8609
  P(IRR > 1.3) = 0.7699
  P(IRR > 1.5) = 0.5477
  P(IRR > 2.0) = 0.1306
  P(IRR < 1.0) = 0.0306
  posterior median IRR = 1.542
  90% credible interval = 1.054 - 2.256
  95% credible interval = 0.980 - 2.427

==============================================================================
Quantity                                     Primary (60 mo) Truncated (72 mo)
------------------------------------------------------------------------------
P(IRR > 1.0)                                           0.849             0.969
P(IRR > 1.2)                                           0.612             0.861
P(IRR > 1.3)                                           0.483             0.770
P(IRR > 1.5)                                           0.265             0.548
P(IRR > 2.0)                                           0.035             0.131
P(IRR < 1.0)                                           0.151             0.031
```

```
Posterior median IRR                                      1.29            1.54
90% credible interval                                0.86-1.92       1.05-2.26
95% credible interval (= Wald CI)                    0.80-2.08       0.98-2.43
==============================================================================
```

Figure S1. Observed monthly series and fitted primary model

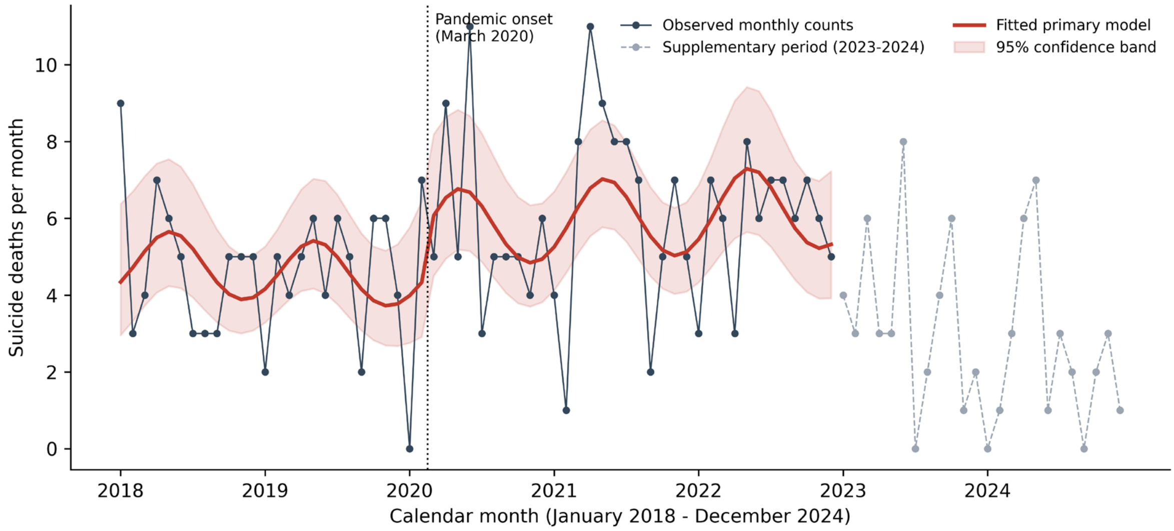

Observed monthly suicide counts for the full 84 months (January 2018 - December 2024), with the supplementary period 2023-2024 distinguished by a broken line, and the fitted values of the pre-specified primary model over its 60-month estimation window with the 95% confidence band superimposed. The vertical dotted line marks the a-priori pandemic onset of March 2020 (month 27 of the series). The figure is drawn at 600 dpi.
